# CHANGES IN MARITAL AND HEALTH STATUS AS RISK FACTORS FOR A SUBSEQUENT NEGATIVE WEALTH SHOCK IN THE US, 1996–2020

**DOI:** 10.1093/geroni/igae098.1273

**Published:** 2024-12-31

**Authors:** Tsai-Chin Cho, Sara Adar, HwaJung Choi, Kenneth Langa, Lindsay Kobayashi

**Affiliations:** University of Michigan, Ann Arbor, Michigan, United States; University of Michigan, Ann Arbor, Michigan, United States; University of Michigan, Ann Arbor, Michigan, United States; University of Michigan, Ann Arbor, Michigan, United States; University of Michigan, Ann Arbor, Michigan, United States

## Abstract

Negative wealth shocks, defined as a loss of ≥75% of one’s wealth over a short time-period, may decrease material living standards in later-life. However, the risk factors for negative wealth shocks are unclear. We aimed to examine the association of four risk factors, 1) separation from or loss of a spouse/partner; 2) diagnosis of a new chronic condition; 3) transition from low to high depressive symptoms; and 4) transition from normal cognition to cognitive impairment or dementia, with the subsequent experience of a negative wealth shock in mid-to-later life. We included respondents aged ≥55 in the US Health and Retirement Study between 1996-2020 (N=128,213 observations from 25,265 persons). Study participants were classified at each time-point as either having experienced a negative wealth shock between each biennial survey interview, or not. The risk factors were assessed over two years preceding negative wealth shocks. We performed multivariable logistic mixed effects models accounting for complex survey design to examine how each risk factor may trigger negative wealth shocks and evaluate effect modification by retirement status at the baseline of study intervals. Separation from or loss of a spouse/partner increased the odds of a negative wealth shock (AOR= 1.44; 95% CI: 1.24, 1.67). Compared to those not retired, retirees who became depressed had higher odds of a negative wealth shock (AOR= 1.23; 95% CI: 1.04, 1.47). This population-based prospective study suggests that changes in marital status and in mental health, for retired adults, may signal future negative wealth shocks, although further investigation is required.

